# Development of Biomarkers for Inhibition of SLC6A19 (B^0^AT1)—A Potential Target to Treat Metabolic Disorders

**DOI:** 10.3390/ijms19113597

**Published:** 2018-11-14

**Authors:** Kiran Javed, Qi Cheng, Adam J. Carroll, Thy T. Truong, Stefan Bröer

**Affiliations:** 1 Research School of Biology, Australian National University, Canberra, ACT 2601, Australia; kiran.javed@anu.edu.au (K.J.); qi.cheng@anu.edu.au (Q.C.); 2 ANU Joint Mass Spectrometry Facility, Research School of Chemistry, Australian National University, Canberra, ACT 2601, Australia; adam.carroll@anu.edu.au (A.J.C.); thy.truong@anu.edu.au (T.T.T.)

**Keywords:** amino acid absorption, epithelial transport, metabolomics

## Abstract

Recent studies have established that dietary protein restriction improves metabolic health and glucose homeostasis. SLC6A19 (B^0^AT1) is the major neutral amino acid transporter in the intestine and carries out the bulk of amino acid absorption from the diet. Mice lacking SLC6A19 show signs of protein restriction, have improved glucose tolerance, and are protected from diet-induced obesity. Pharmacological blockage of this transporter could be used to induce protein restriction and to treat metabolic diseases such as type 2 diabetes. A few novel inhibitors of SLC6A19 have recently been identified using in vitro compound screening, but it remains unclear whether these compounds block the transporter in vivo. To evaluate the efficacy of SLC6A19 inhibitors biomarkers are required that can reliably detect successful inhibition of the transporter in mice. A gas chromatography mass spectrometry (GC-MS)-based untargeted metabolomics approach was used to discriminate global metabolite profiles in plasma, urine and faecal samples from SLC6A19ko and wt mice. Due to inefficient absorption in the intestine and lack of reabsorption in the kidney, significantly elevated amino acids levels were observed in urine and faecal samples. By contrast, a few neutral amino acids were reduced in the plasma of male SLC6A19ko mice as compared to other biological samples. Metabolites of bacterial protein fermentation such as p-cresol glucuronide and 3-indole-propionic acid were more abundant in SLC6A19ko mice, indicating protein malabsorption of dietary amino acids. Consistently, plasma appearance rates of [^14^C]-labelled neutral amino acids were delayed in SLC6A19ko mice as compared to wt after intra-gastric administration of a mixture of amino acids. Receiver operating characteristic (ROC) curve analysis was used to validate the potential use of these metabolites as biomarkers. These findings provide putative metabolite biomarkers that can be used to detect protein malabsorption and the inhibition of this transporter in intestine and kidney.

## 1. Introduction

Protein restriction is now recognized as an important factor for the maintenance of metabolic health. This is supported by several studies suggesting that dietary protein is the major mediator of benefits achieved by dietary restriction [1,2,3]. Moreover, Newgard et al. [4] showed a strong correlation between the risk of developing type 2 diabetes and increased plasma levels of essential amino acids (isoleucine, leucine and valine), aromatic amino acids (phenylalanine and tyrosine) and their metabolites such as C3 and C5-acyl carnitines. These amino acids have a prognostic power for future development of type 2 diabetes [5,6] and insulin resistance [7]. Recent work published by Fontana et al. [8] showed that reduction in body mass and improved glucose tolerance can be achieved by only restricting branched-chain amino acids (BCAA) in the diet in young mice. Cummings et al. [9] extended those studies to obese mice and showed that a low BCAA diet was more effective in weight loss and improving glucose tolerance than low fat diet [10]. To test whether BCAA restriction is necessary to achieve effects of protein restriction, Maida et al. [11] replenished BCAA in a protein-restricted diet in wild type (wt) and New Zealand obese (NZO) mice and found that the metabolic effects of protein restriction was reversed in wt mice but not in obese mice. This suggested that that BCAA restriction alone is not sufficient to account for all the effects of protein restriction [12]. Restriction of methionine, for instance, has also been reported to underlie some of the effects of calorie restriction [13,14], most likely involving the general control non-derepressible-2 (GCN2) pathway and epigenetic mechanisms [15,16]. A key hormone involved in the metabolic effects of protein restriction appears to be fibroblast growth factor 21 (FGF21) [10,17,18]. Although FGF21 transcription is upregulated under conditions of protein restriction, its metabolic actions are largely restricted to lipid metabolism, such as reduction of plasma lipids and production of adiponectin and ketone body biosynthesis [19]. 

However, amino acids are signalling molecules in their own right and there are several signalling pathways, such as mammalian target of rapamycin (mTORC1) and GCN2 that closely monitor amino acid levels in the cytosol and lysosomes [20]. Of the 20 proteinogenic amino acids certain groups have been highlighted as signalling molecules in protein restriction. BCAA, for instance, activate mTORC1 and show a decline after therapeutic interventions such as gastric bypass surgery [21]. Gastric bypass surgery uses a combination of restriction and malabsorption of nutrients, which results in dramatic weight loss, improvement of hyperglycaemia [22] and causes remission of type 2 diabetes in the obese patients [23]. Methionine is an important methyl-group donor and restriction of this amino acid has also shown to generate similar effects as protein restriction in general [14].

The major mediator for the absorption of BCAA and methionine in the intestine is the neutral amino acid transporter SLC6A19 (solute carrier family 6 member 19; B^0^AT1). SLC6A19ko mice show a metabolic phenotype similar to that achieved by protein restriction such as improved glucose tolerance, reduced body weight, elevated FGF21, browning of white adipose tissue, reduced plasma lipids [24]. In addition to FGF21 [10], SLC6A19ko mice also show enhanced production of glucagon like peptide 1 (GLP-1) after nutrient intake. This is caused by reduced absorption of amino acids, resulting in a higher luminal load of neutral amino acids, which act as signalling molecules to release GLP-1 from enteroendocrine cells [25]. FGF21 and GLP-1 are currently being used for the treatment of diabetes or are in clinical trials [26,27]. SLC6A19 is also present in the early sections of the kidney proximal tubule where it reabsorbs amino acids back into the blood from the ultra-filtrate generated in the glomeruli of the kidney cortex. Because of the role of this transporter in the homeostasis of BCAA and methionine levels, we have postulated that pharmacological inhibition of the transporter may induce similar metabolic effects as protein restriction and could be used as a treatment for metabolic diseases [28]. The transporter has also been suggested as a target to treat specific disturbances of amino acid homeostasis such as phenylketonuria [29].

Mutations in SLC6A19 are found in Hartnup disorder patients, which is diagnosed by the spillover of neutral amino acids in the urine [30]. The disease is largely benign, and hence it is likely that pharmacological inhibition can be achieved without detrimental side-effects on human health. As a result, attempts have been made to identify inhibitors of SLC6A19 using in vitro assays [31,32]. However, it is essential to determine whether inhibitors will be able to block both intestinal and renal transport of amino acids in vivo where competing substrates are present. The purpose of this study was to predict inhibition of SLC6A19 from biomarkers in biological samples, such as urine, plasma and faeces. As a surrogate for inhibition of SLC6A19 we compared wt mice with global SLC6A19ko mice. We employed untargeted metabolomics using gas chromatography mass spectrometry (GC-MS) to screen for biomarkers in readily accessible metabolic samples [33,34]. 

## 2. Results

**Figure 1:**
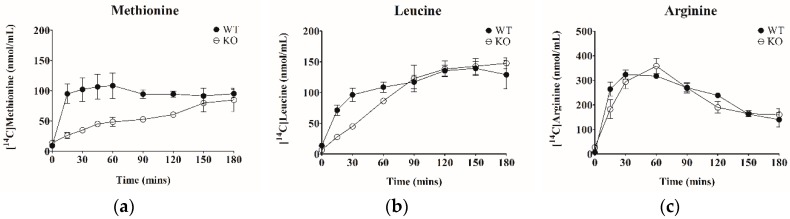
Absorption of amino acids in mouse intestine. Mixtures of unlabelled amino acids and specific radiolabelled amino acids were offered to mice as jelly food. Plasma concentration–time profiles of [14C]-methionine (**a**), [14C]-leucine (**b**) and [14C]-arginine (**c**) in SLC6A19ko (○) and SLC6A19wt (●) mice. Data are expressed as mean ± S.E. (*n* = 4).

SLC6A19 is the major transporter for neutral amino acids in the small intestine. To demonstrate the role of the transporter in amino acid absorption in the intestine, the appearance of radiolabelled amino acids in blood plasma after oral administration of food jelly containing radiolabelled amino acids was measured (*n* = 4). As shown in Figure 1, the appearance of [^14^C]-leucine and [^14^C]-methionine in blood plasma was substantially delayed in SLC6A19ko mice as compared to wt (*p* < 0.01). Over a time course of 3 h, however, methionine and leucine were absorbed, suggesting redundant capacity for amino acid absorption. The area under curve (AUC) of [^14^C]-arginine absorption, by contrast, was not different between wt and SLC6A19ko mice, consistent with arginine not being a substrate of SLC6A19.

We hypothesized that reduced intestinal amino acid absorption in SLC6A19ko mice resulted in increased levels of amino acids in the lumen of the intestine and progression of amino acids into more distal sections of the intestine, where the majority of the microbiome is located. Consistent with increased microbial metabolism of amino acids, faecal samples of SLC6A19wt mice had lower levels of ammonia than SLC6A19ko (p-value 0.01) (Table 1). No differences were observed in plasma and urine, most likely due to the efficient removal of ammonia by the liver. When the intake of amino acids exceeds the needs for net protein biosynthesis, amino acids are used as energy metabolites, which requires deposition of amino groups as urea. Consistent with signs of protein restriction, urea levels were reduced in the plasma of SLC6A19ko mice as compared to SLC6A19wt mice (Table 1). 

### 2.1. Overview of Untargeted Metabolomics of SLC6A19ko and SLC6A19wt Mice

**Figure 2:**
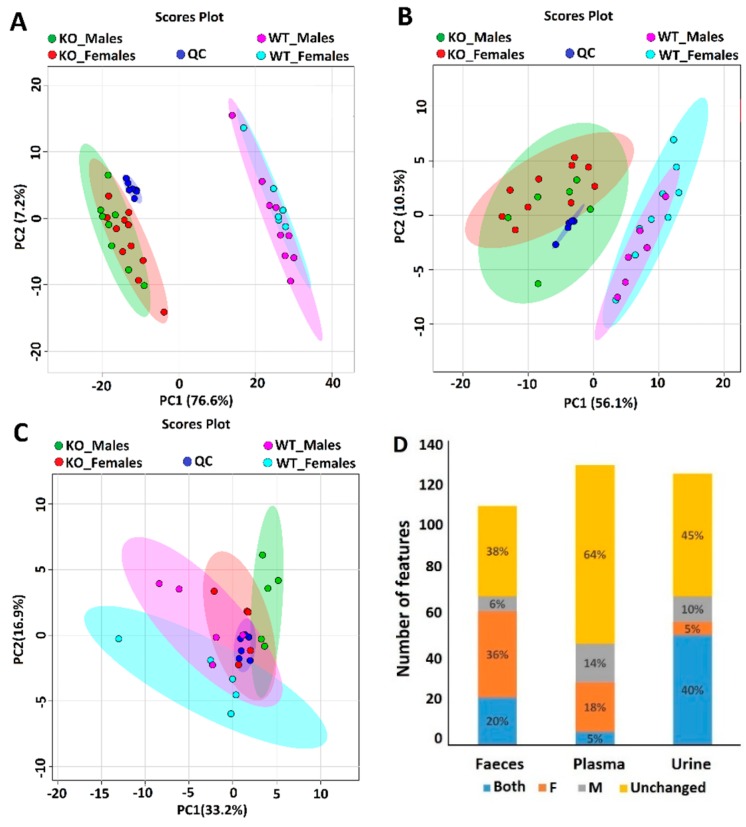
Principal component analysis of urine (**A**), faeces (**B**), and plasma (**C**) metabolome in SLC6A19ko and SLC6A19wt mice. Red data points represent ko females, green data points represent ko males, dark blue data points show quality control (QC) samples, light blue data points show wt females and pink data points wt males. Principal component analysis (PCA) separates the samples by genotype in urine (**A**) and faeces (**B**), but not in plasma (**C**). (**D**) Stack plot showing metabolic features that have different abundance in wt and ko mice regardless of gender as indicated by blue. Metabolic features that are unchanged are shown in yellow. Features only showing differences between wt and ko females are shown in orange, those that are different in males only are shown in grey. The y-axis shows the total number of metabolic features found in the samples.

To determine if we could predict the lack (inhibition) of SLC6A19 in kidney and intestine, we determined the urinary, faecal and plasma metabolome using GC-MS. This untargeted method was able to detect 126, 130 and 111 metabolic features with percent relative standard deviation (RSD < 40%) in urine, plasma and faeces, respectively. Metabolic features are molecules that have gone through hard ionization via electron impact (EI, 70 eV) to produce characteristic MS spectra of positive mass-to-charge (*m/z*) ion fragments in the ion source of the mass spectrometer. The MS spectrum for each molecule is matched to established mass spectral databases for identification. Positively identified metabolites will be discussed later. Quality control (QC) samples were included to evaluate the stability of the samples and to assess the reproducibility of the GC-MS performance throughout the batch sequence analysis. The tight clustering of the QC samples showed good stability and reproducibility of the GC-MS method. Principal component analysis (PCA) was statistically used to compare the data of urine, faecal, plasma and QC samples (Figure 2). 

Consistent with previous data [35] unsupervised PCA of urine samples showed a clear separation between SLC6A19ko and SLC6A19wt mice (Figure 2A). Most of the variation (76.6%) was captured in the first principal component (PC1), which suggested that the metabolic profile was intensely perturbed by the influence of SLC6A19. Genotype had a far bigger impact on the urine metabolic profile than gender difference. As summarized in Figure 2D, univariate analysis of urine samples showed differential abundances of about 51 metabolic features. Of the total metabolic features 40% were different between SLC6A19ko and SLC6A19wt mice irrespective of the gender (>2-fold change; *p* < 0.05). These features were largely associated with neutral amino acids (see below). Only 6 (5%) and 12 (10%) metabolic features were specific to females and males, respectively. The metabolic profile of faecal samples of SLC6A19ko mice clustered separately from wild type mice, as shown in Figure 2B, although the separation was less prominent than that observed in urine samples. Females showed a higher number of metabolic features that changed in abundance (36%) as compared to males (6%) (Figure 2D). Twenty-two metabolic features were different between SLC6A19ko and SLC6A19wt mice regardless of gender. The metabolomics profile of plasma samples (Figure 2C) showed the least difference between female SLC6A19ko and SLC6A19wt samples, whereas PCA was able to separate male SLC6A19ko from male SLC6A19wt. The univariate analysis showed that most of the features were unchanged in plasma samples. Only 6 (5%) metabolic features were found to be expressed differently regardless of gender, whereas 23 features (18%) were different between SLC6A19ko and SLC6A19wt females and 18 (14%) were different between SLC6A19ko and SLC6A19wt males (Figure 2D). 

### 2.2. Metabolic Profiling in Urine Samples

**Figure 3:**
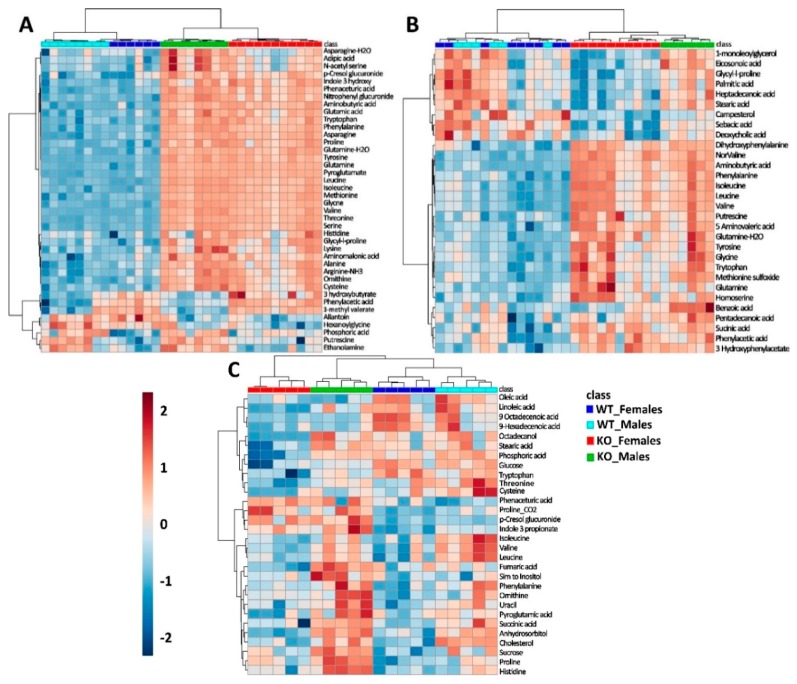
A two-way hierarchal cluster analysis (HCA) and heat map of metabolite differences in urine (**A**), faeces (**B**) and plasma (**C**) of SLC6A19ko and SLC6A19wt mice. Unsupervised HCA and heat map of metabolites demonstrates the separation of SLC6A19ko and SLC6A19wt mice in urine (**A**), faeces (**B**) and plasma (**C**). Red represent ko females, green represents ko males, dark blue shows wt females and light blue shows wt males. The tree diagram shows dendograms of hierarchal clustering (Minkowsky’s method). Colours indicate the relative concentration of each metabolite after log transformation and every column represents one sample.

All metabolic features were annotated using reference library databases such as the Golm Metabolome Database (GDM) and the National Institute of Standards and Technology (NIST) with Kovats non-isothermal retention indices (RI). For this study, unknown features were removed from the dataset. Among the annotated features, the top 50 candidates were selected and plotted as a hierarchal cluster analysis (HCA) and heatmap (Figure 3A). SLC6A19ko mice clustered together in the HCA dendogram according to their gender (red/blue vs. green/light-blue) and genotype (red/green vs. blue/light-blue) indicating a strong discrimination of the metabolome in urine samples. A list of metabolites that are either up-regulated or down-regulated significantly depending on genotype and gender are listed in Table 2. 

Consistent with previous results [35,36] neutral amino acids such as serine, cysteine, aminobutyric acid, leucine, isoleucine, valine, threonine, asparagine, glutamine, methionine, tyrosine, tryptophan and phenylalanine, showed the largest difference between SLC6A19ko and SLC6A19wt mice. Even poor substrates of SLC6A19 such as proline, glycine, increased in abundance in the urine of SLC6A19ko mice, suggesting limited redundancy. We also found significantly higher levels of glutamic acid and lysine in the urine of SLC6A19ko mice, possibly pointing to downregulation of other transporters in the kidney. However, comparison of microarray data did not reveal significant expression changes of transporters responsible for the absorption of these substrates such as SLC7A7, SLC7A9, SLC3A1, or SLC1A1 in the kidney of SLC6A19ko mice (Table 3). A few bacterial metabolites were also increased in abundance in SLC6A19ko mice, such as p-nitrophenyl-galacturonide (35.02-fold), p-cresol glucuronide (8.8-fold), 5-hydroxy-indole (5.9-fold increase), phenaceturic acid (14-fold increase) and 3-hydroxyphenylacetic acid (1.6-fold increase). An opposite trend was shown by hexanoylglycine and butrylglycine that were less abundant in SLC6A19ko mice than in the wt. Most of the metabolites showed a slightly more prominent change among males than in females. Taurine, succinate, thymine, allantoin and quinolone-2-carboxylic acid were downregulated in males but did not reach significance in females.

### 2.3. Metabolic Profiles in Faecal Samples

The global deletion of SLC6A19 leads to the significant loss of amino acids through faeces as well. Figure 3B displays the abundance of metabolites in faecal samples of SLC6A19ko and SLC6A19wt mice as a two-way HCA and heat-map. We observed increases of neutral amino acids such as leucine, isoleucine, valine, phenylalanine, tryptophan, tyrosine and threonine in the faecal samples in both genders of SLC6A19ko mice. This confirms a major role of the transporter for neutral amino acid absorption in the intestine. Moreover, bacterial metabolites of different amino acids were found to be elevated, such as 5-amino-valeric acid, 3-hydroxybenzeneacetic acid, tyramine, dihydroxyphenylalanine, 4-hydroxyphenylacetic acid, 5-hydroxy-indole and putrescine (Table 4). Reduced abundance in faecal samples of SLC6A19ko was noticed in the case of deoxycholic acid and sebacic acid in females. 

### 2.4. Metabolic Profiles of Plasma Samples

Consistent with our previous results [24], we could not identify significant differences of plasma metabolite levels in SLC6A19ko and SLC6A19wt mice under fasting conditions. As a result, we performed untargeted metabolomics analysis of plasma samples under postprandial conditions. While we were able to separate mice by genotype with the help of hierarchal cluster analysis (Figure 3C), the differences were not as profound as in urine or faecal samples. The metabolic profile of males was quite different from females, which shows that the effect of gene deletion is gender dependent and that gene deletion effects are rather small (Table 5). For instance, lipid metabolites were significantly changed in female SLC6A19ko compared to female SLC6A19wt mice, but did not reach significance in males. Similarly, we found reduced levels of BCAA in male SLC6A19ko mice (FC 0.78; p value 0.08); however, BCAA levels remain unchanged in female SLC6A19ko mice as compared to *wt*. Tryptophan, by contrast, was markedly reduced in female SLC6A19ko mice (FC 0.4; *p* = 0.001). We observed increased levels of proline, lysine and histidine in the plasma of SLC6A19ko mice. Bacterial amino acid metabolites showed a significant upregulation irrespective of the gender. For instance, tryptophan metabolite indole-3-propionic acid increased 4.4-fold (*p* = 0.001) and p-cresol glucuronide, a bacterial metabolite originating from aromatic amino acids increased by 17.34 folds (*p* < 0.001) in SLC6A19ko samples. Fatty acids such as stearic acid, linoleic acid and palmitic acid were reduced in female SLC6A19ko mice as compared to wt. 

Identification of amino acids and primary metabolites was confirmed with authentic standards in this study (shown in Table 3, Table 4 and Table 5). While we did not have standards for bacterial metabolites their Kovats RI and reverse spectral match was >850 with probabilities >90%. A comparison of the mass spectra of indole-3-propionic acid and p-cresol-glucuronide with that of the NIST reference spectra are shown in the Figure S1.

### 2.5. Assessment of Biomarkers for the In Vivo Detection of SLC6A19 Inhibition

Receiver operating characteristic (ROC) curve analysis was used to identify biomarkers that can be used reliably to detect lack of SLC6A19 in kidney and intestine. The sensitivity and specificity were calculated for each biomarker using the area under receiver operator characteristic curve (AUROC). A biomarker with an AUC of 1 would have the perfect ability to predict the inhibition of SLC6A19, whereas an AUC of 0.5 would have no predictive power. Table 6 enlists the AUC analysis of metabolites present in urine, faeces and plasma of SLC6A19ko and SLC6A19wt mice. Lack of SLC6A19 in the kidney is readily detected through 38 metabolites that have an AUROC > 0.8 in urine samples. Almost all neutral amino acids performed well (Table 6) with *p* < 0.001. In faecal samples, 30 metabolites showed an AUROC > 0.8 and in plasma only 20 metabolites exhibited an AUROC > 0.8. Neutral amino acids did not perform well in plasma samples. The best predictors of SLC6A19 inhibition in plasma samples were indole-3-propionic acid, (AUROC 0.99 with *p* = 0.013) and p-cresol glucuronide (AUROC 0.99; *p* = 0.004). Indole-3-propionic acid also showed high positive correlation with p-cresol-glucuronide (AUROC 0.85; *p* = 1.8 × 10^−6^) and phenaceturic acid (0.62; *p* = 0.003) (data not shown).

**Figure 4:**
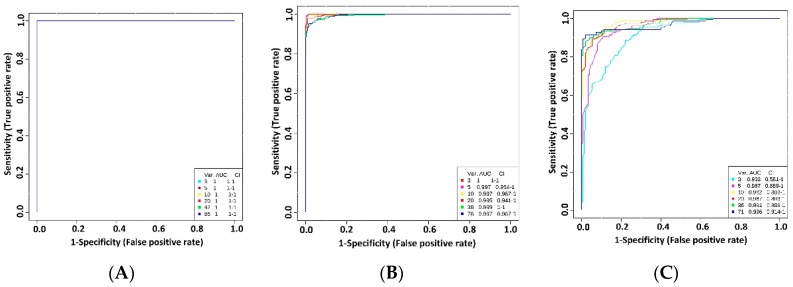
Performance of biomarkers in biological samples. Urine (**A**), faecal (**B**) and plasma (**C**) samples were taken from SLC6A19ko and SLC6A19wt mice. Univariate AUROC was used as classification and feature ranking method. In each Monte-Carlo Cross Validation, two-thirds of the samples were used to evaluate the importance of the metabolite. Different panels of important features were then used to build classification models, which were validated on the remaining samples. The different models with specific feature numbers written on the bottom right of the figure and their corresponding AUCs are shown on the figure. Var. (variables) indicates the number of selected metabolites.

Because individual metabolites may fail to predict the correct genotype, we investigated combination of metabolites as potential biomarkers in urine, faecal and plasma samples (Figure 4). ROC curves were generated through Monte-Carlo cross validation in which two thirds of the samples were used to build a model, which was then used to predict the samples that were left out. Neutral amino acids, individually as well as in combination, had strong predictive power according to multivariate ROC curves in urine and faecal samples as shown in Figure 4A,B. In plasma samples (Figure 4C), the best model consisting of the top 5 metabolites (indole-3-propionic acid, tryptophan, cresol-glucuronide, ornithine, 11-octadecanoic acid) showed an AUROC value of 0.987 (95% C.I. 0.889-1) after which no improvement was seen in the AUC curve by further adding more metabolites. Together, these results suggest that these biomarkers, alone or in combination, have the potential of reliably identifying inhibition of SLC6A19 in kidney and intestine.

### 2.6. Titration of SLC6A19 Transporter Activity 

It is essential to know if biomarkers are linearly correlated with the transport activity. To evaluate the correlation of inhibition of SLC6A19 with the biomarker abundance, we analysed samples from heterozygous (hz) mice in addition to SLC6A19ko and SLC6A19wt samples (*n* = 4). The heterozygous mice expressed approximately 50% of the protein as shown previously [35]. 

**Figure 5:**
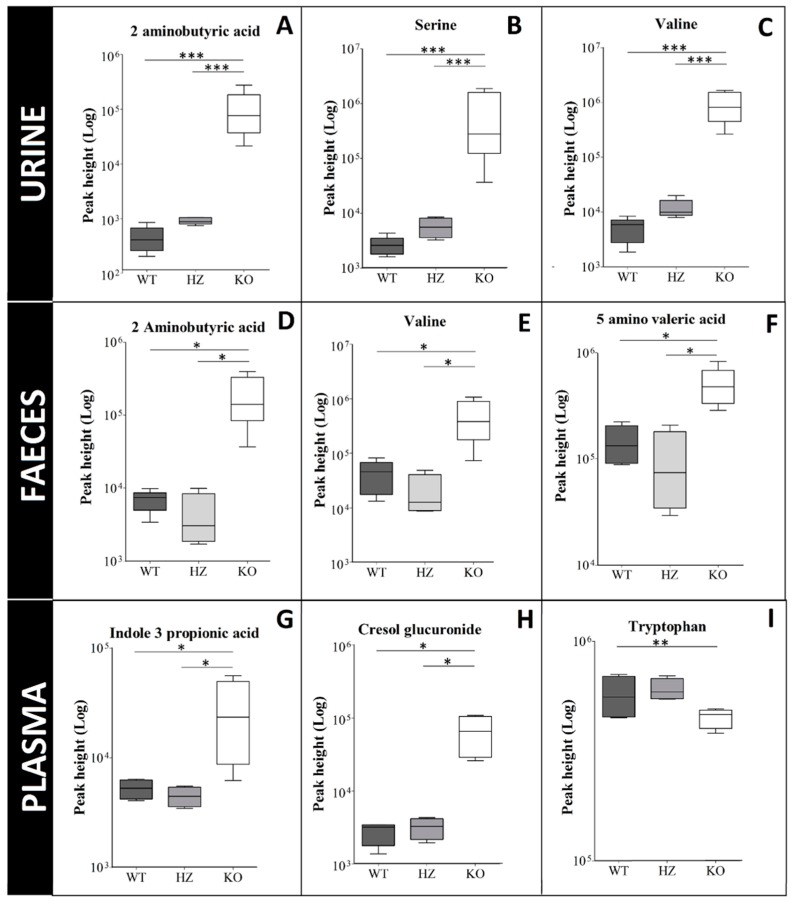
Correlation between SLC6A19 expression and biomarker levels. Box plots of individual biomarkers in SLC6A19 ko, hz and wt samples. Urine metabolites: (**A**) 2-aminobutyric acid, (**B**) serine, (**C**) valine; faecal metabolites: (**D**) 2-aminobutyric acid, (**E**) valine, (**F**) 5-amino valeric acid; plasma samples: (**G**) indole-3-propionic acid, (**H**) cresol glucuronide, (**I**) tryptophan. Horizontal lines represent from bottom to top: the minimum, 25th, 50th, and 75th percentiles and the maximum. Solid line within the boxplot represents median value. Asterisks define the p value; ***: *p* < 0.001, **: *p* = 0.001 to 0.01 and *: *p* = 0.01 to 0.05.

This analysis shows that biomarkers did not show a linear trend in response to inhibition of SLC6A19. Figure 5 only shows selected biomarkers plotted individually for each biological matrix. Haploinsufficiency of SLC6A19 in kidney causes a slight increase in abundances of all neutral amino acids in urine, but the trend was not linear. Biomarkers selected in plasma and faecal samples did not show any increase in abundance in heterozygous mice. This result is consistent with the recessive phenotype of Hartnup disorder in humans.

### 2.7. Evaluation of Biomarkers In vivo

**Figure 6:**
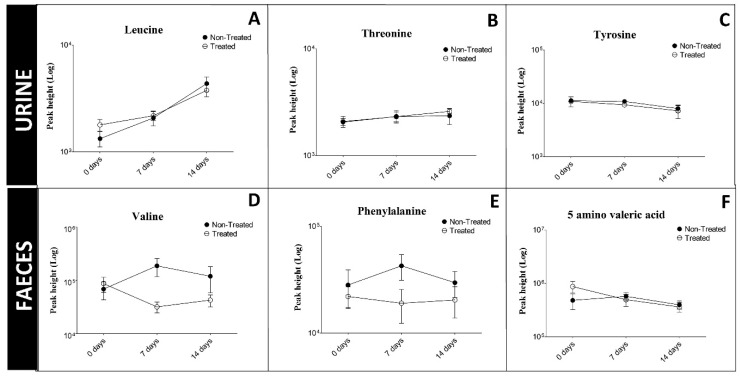
Changes of urinary biomarkers (**A**) leucine, (**B**) threonine, (**C**) tyrosine and faecal biomarkers (**D**) valine, (**E**) phenylalanine, (**F**) 5 amino valeric acid during treatment of animals with benztropine. Female C57BL/6J mice were provided with chow containing 1.25 mg benztropine/5 g or received normal chow. Urine and faecal samples were taken before treatment and after 7 and 14 days of treatment.

We previously identified benztropine as a competitive inhibitor of SLC6A19 (B^0^AT1) [31]. Competitive inhibitors can be displaced by the physiological substrate of the transporter and we therefore tested the efficacy of benztropine in vivo. We fed mice 1.25 mg benztropine per 5 g chow per day over a period of two weeks, a dose that is tolerated by mice [37]. At this dose we did not observe an increase of biomarkers in urine or faeces in treated mice as compared to untreated mice (Figure 6). The results indicate that benztropine provided at a pharmacologically active dose, does not inhibit SLC6A19 to an extent that did raise biomarkers.

## 3. Discussion

Protein restriction is an increasingly attractive approach to treat metabolic disorders that are associated with insulin resistance and obesity. Genomic deletion of SLC6A19 in mice improves the metabolic health as indicated by parameters such as better glucose tolerance, lower plasma and liver triglycerides [24], and improved insulin sensitivity [38]. Whether similar beneficial effects are observed in humans with mutations in SLC6A19 remains to be shown. To test the efficacy of known [31,32] or novel B^0^AT1 inhibitors, SLC6A19ko mice were used as a surrogate to identify diagnostic biomarkers. The absence of SLC6A19 in the kidney is characterized by the loss of amino acids in urine, a hallmark of Hartnup disorder. This is a consequence of reduced reabsorption of amino acids in the kidney proximal tubule [30]. However, the absence of SLC6A19 in the intestine has not been linked to a metabolic phenotype as yet. In this metabolomics study, we separated gender into different classes whereas age was kept constant to improve the robustness of biomarkers. We identified biomarkers that can detect the inhibition of SLC6A19 in the intestine specifically through analysis of faecal matter, but also through the detection of bacterial amino acid metabolites in blood plasma and urine. Urine, plasma and faeces were selected as the biological matrices for profiling biomarkers because they can be easily obtained and analysed rapidly. Not unexpectedly, the lack of SLC6A19 was most prominently observed in samples from urine and faeces.

A combination of proteases, peptidases, peptide and amino acid transporters ensures that 95% of dietary protein intake is digested and absorbed [39]. After absorption, amino acids are distributed to all organs. Lack of SLC6A19 causes protein malabsorption and bio-distribution of amino acids to tissues is delayed. Our results and the results of other groups demonstrate that impaired amino absorption causes an increased delivery of these metabolites to the distal intestine and subsequently increased levels of amino acid fermentation products are produced by the colonic microflora [40,41]. These fermentation products appear to be the most reliable biomarkers to predict the inhibition of SLC6A19 in the intestine because they increase on a low background. Biomarkers of bacterial origin have also been observed in Hartnup disorder differentiating it from purely renal amino acidurias [42]. Our study confirms that genomic deletion of SLC6A19 causes significant changes in the metabolic profile of urine and faeces, and in addition identifies several biomarkers with diagnostic value, particularly when used in combination. The key diagnostic urinary and faecal metabolites were neutral amino acids, i.e., the substrates of the transporter. In plasma samples the key diagnostic biomarkers increased indole-3-propionic acid and p-cresol-glucuronide. Of the SLC6A19 substrates only tryptophan levels were found to be reduced in blood plasma. In agreement with glucose tolerance tests [24] postprandial glucose levels were reduced in SLC6A19ko mice. It is important to note that these biomarkers are not linearly correlated with transport activity. In heterozygous animals no reliable biomarkers could be identified in any biological matrix, suggesting that biomarkers only appear when transport is strongly inhibited. One possibility to further investigate this non-linear correlation in future studies is to vary the protein content of the chow and evaluate the responses in heterozygote and knock-out animals.

The most likely explanation of increased neutral amino acids in the faecal samples of ko mice is the malabsorption of amino acids in the intestine, resulting in their excretion through faeces. However, it could also be a result of changes to the abundance or a shift in the composition of microbiota due to dietary factors. Gut microbiota are known to synthesize BCAA from pyruvate and ketobutyrate and therefore may contribute to amino acid load in faecal samples [43]. Analysis of the microbiome in Angiotensin converting enzyme 2 (ACE2) knock out mice, which lack SLC6A19 protein in the intestine, shows increased abundance of gut microbiota as compared to wild type mice [44]. It is not quite clear why bacterial metabolites of aromatic amino acids stand out as biomarkers. Possibly, fermentation products of other amino acids are more easily absorbed by the intestine. For instance, valeric acid and caproic acid may be more readily absorbed than 4-hydroxyphenylacetic acid. Dietary tyrosine and phenylalanine are metabolized into 4-hydroxyphenylacetic acid and then into p-cresol mainly in the distal small intestine by anaerobic bacteria [45]. After absorption they are conjugated to glucuronide or sulphate for urinary excretion [46]. Accordingly, we observed higher levels of p-cresol glucuronide in plasma as well as urine of SLC6A19ko mice. p-cresol itself is volatile and is likely to disappear in the lyophilisation process associated with the preparation of faecal samples. However, we found the less volatile 4-hydroxyphenylacetic acid in faecal samples of ko mice. Previously, we have also reported increased levels of bacterial fermentation products of leucine and isoleucine in the faecal samples of knockout mice in a targeted metabolomics study [24]. The malabsorption of dietary amino acids increased the abundance of these metabolites in the intestine, mimicking that of a high protein load. This is consistent with a positive correlation of colonic production of p-cresol with dietary protein intake [47]. Interestingly, increased levels of p-cresol have also been reported after bariatric surgery in humans [48] and rats [49] in which nutrient malabsorption occurs. It has been speculated that some of the resulting metabolites contribute to metabolic changes observed after surgery [50]. However, the metabolic phenotype of an organism is sensitive to diet, age, gender and other intrinsic and extrinsic factors [51,52]. Plasma samples showed only slight changes in the metabolic profile between SLC6A19ko and SLC6A19wt mice. The concentration of amino acids in the plasma are tightly regulated by mechanisms involving production/influx or consumption/efflux of amino acids [20]. The production/influx of amino acids is influenced by dietary intake, tissue protein degradation in muscle and microbial synthesis of amino acids [53], whereas the consumption/efflux of amino acids is a result of protein synthesis, excretion through urine or faeces and catabolism/oxidation in the body [54]. We observed only a slight reduction in BCAA in SLC6A19ko males whereas no changes were observed in females. It has been observed before that plasma concentration of amino acids varies more in men as compared to women after weight loss [55] or protein restriction in diet [56]. As evidenced by reduced urea levels, amino acid catabolism is down-regulated in SLC6A19ko mice. Moreover, enhanced degradation of protein in skeletal muscle further compensates for the loss of amino acids through excretion and malabsorption [24]. 

In conclusion, with the help of untargeted metabolomics using GC-MS, we identified neutral amino acids as the most reliable biomarkers of SLC6A19 inhibition in urine and faeces. These can be used as biomarkers to evaluate inhibition in the kidney and intestine. In addition, gut-derived bacterial metabolites of amino acids such as p-cresol-glucuronide and indole-3-propionic acid can be used as plasma biomarkers to assess inhibition of intestinal amino acid transport. Our metabolomics approach showed us reliable biomarkers that can be used further for screening inhibitors of SLC6A19 effectively. Our biomarkers suggest that benztropine, used at a pharmacologically active dose in vivo [37], did not inhibit SLC6A19 strongly enough to raise the level of biomarkers. Further screening and compound optimization is required to produce potent in vivo inhibitors.

## 4. Materials and Methods 

### 4.1. Animals and Sample Collection

SLC6A19ko and SLC6A19wt littermates were used in the experiments. The metabolic phenotype of these mice has been described previously [24,35]. All mice were 4–6 months of age, male and female mice were analysed separately to account for gender differences. As reported previously, SLC6A19ko mice have reduced body weight as compared to their wildtype controls (Table 1). Animals were fed with a standard rodent diet (Table S1). For sample collection, mice were acclimatized to the metabolic cages (Techniplast, Buguggiate, VA, Italy) for 2 days before 18h sample collection of urine and faecal samples. In the metabolic cages, mice had free access to water only, because food debris can contaminate the sampling procedure. To avoid excessive calorie restriction, mice were placed back in their cages after every 8 h for an h allowing food consumption. All animal experiments were approved by the animal experimentation ethics committee of the Australian National University (Protocol: A2013/39 (approved on 12 May 2016) and A2016/41 (approved on 29 November 2016). The collection tube was placed in dry ice during the collection time to quench any enzymatic reactions in order to preserve the metabolic profile of the sample. Mice were culled by cervical dislocation and the blood samples were collected in the fed state, consistent with previous results showing metabolic effects of SLC6A19 deficiency mainly after nutrient intake [24]. The plasma samples were isolated in ethylene diamine tetra acetic acid kEDTA tubes (Sarstedt, Nümbrecht, Germany) and all samples were stored at −80 °C immediately. 

### 4.2. Pharmacological Animal Studies

Twelve C57BL/6J female mice (age 6–8 months) were randomly assigned to treatment group and control group. Based on previous study [57] on food and water intake in 28 mouse strains, the daily food intake for C57BL/6 strain is approximately 4–5 grams per mouse. Benztropine was reported to be used at a maximal dosage of 250 mg/kg diet on mice in a study of energy expenditure [37]. Accordingly, 1.25 mg benztropine in 5g of chow was given to each mouse every day. To administer the drug, benztropine mesylate powder (SML0847, ≥98%; Sigma-Aldrich, St. Louis, MO, USA) was dissolved in ddH_2_O at a concentration of 1.25 mg per 250 μL water and soaked into 5 g chow. The fortified pellets were given for 14 days. Each mouse in the control group was provided with same amount of chow soaked in 250 μL water. The prepared chow pellets were stored at 4 °C and pre-warmed to 37 °C before providing them to mice. The consumption of chow pellet was recorded to calculate the daily drug dose.

### 4.3. Radioactive Uptake Assay 

To investigate gastric emptying and absorption of amino acids, 1μCi of [^14^C]-amino acids (Perkin Elmer Life and Analytical Sciences, Waltham, MA, USA) such as methionine, leucine and arginine were incorporated into jelly containing an unlabelled amino acid mix (1 g/kg body weight) [58]. The amino acid mixture represents the ratio of amino acids in a standard laboratory diet as listed in Table S2. Mice were trained to eat the un-infused jelly voluntarily within 5 minutes on the day before the experiment. On the day of the experiment, mice were fasted for 16 h before the presentation of the jelly containing labelled and unlabelled amino acids. Only mice that consumed all the jelly within 5 min of presentation were analysed. A blood sample of 10 μL was taken every 30 min for approximately 3 h. The blood samples were allowed to clot and then centrifuged at 13,400× *g* for 2 min to obtain serum. The amount of [^14^C]-amino acids were then quantified using a Microbeta trilux scintillation counter (Perkin Elmer Life and Analytical Sciences, Waltham, MA, USA).

### 4.4. Ammonia and Urea Assay

Plasma and urine samples were diluted 1:10 and analysed for the measurement of ammonia levels using an ammonia assay kit. The manufacturer’s instructions were followed with small modifications (AA0100; Sigma-Aldrich, St. Louis, MO, USA). To analyse faecal samples, 50 mg mouse droppings were dissolved in 1000 µL of water and centrifuged at × *g* for 5min to remove cell debris. Then 20 μL of the supernatant was added to 200 μL of Ammonium Assay buffer and baseline absorbance was taken at 340 nm. Subsequently, 2 μL of glutamate dehydrogenase was added to each sample and incubated at room temperature for 10 min. The absorbance at 340 nm was then recorded. Ammonia levels were calculated as described in the protocol. For the urea assay, urine was diluted 1:50 fold and faecal samples were prepared as described above. Plasma was assayed directly using the urea assay kit (Z5030016; Bio chain, Newark, CA, USA).

### 4.5. Untargeted Metabolomics of Urine, Faecal and Plasma Samples

Samples for untargeted metabolomics were prepared using previously established metabolomics methods [59] with small modifications. Urine samples were first centrifuged at × *g* for 5 min to precipitate cell debris and their osmolality was measured. All samples were normalized by osmolality prior to extraction to ensure that equivalent concentrations of metabolites were analysed [60]. The samples were then treated with 50 units of urease at 37 °C for 30 min to deplete highly abundant urea in urine. After centrifugation, methanol: water (9:1, *v*/*v*) was used to inactivate urease and to extract metabolites. Ribitol (100 ng/μL, Sigma-Aldrich, St. Louis, MO, USA) was added as an internal standard. Samples were vortexed at 2500 rpm for 10 min using multi plate shaker (Biosan, Riga, Latvia) and then centrifuged at × *g* for 10 min to pellet insoluble material. A 70 μL aliquot of supernatant was dried down in a speedvac (Labconco, Kansas City, MO, USA) at room temperature for 4 h. Faecal samples were lyophilized for 24 h and pulverized in a TissueLyser bead mill (Qiagen, Hilden, Germany) for 90 s at 20 Hz. A 20 mg sample of faeces was weighed and extracted with HPLC-grade methanol: chloroform (9:1, *v*/*v*). Ribitol (200 ng/μL) was added as an internal standard. Samples were vortexed at 2500 rpm for 10 min and then centrifuged at × *g* for 10 min to pellet insoluble material. Centrifugation was repeated to ensure complete removal of insoluble material before 30 μL of the supernatants were dried in an autosampler vial with a 300 µL fixed insert (Agilent Technologies, Palo Alto, CA, USA) in a speedvac for 3 h. Plasma samples were treated with HPLC-grade methanol/chloroform/Milli-Q water (5:2:2, *v*/*v*/*v*). Ribitol (100 ng/μL) was added as an internal standard. Samples were vortexed at 2500 rpm for 10 min and then centrifuged at × *g* for 10 min to pellet insoluble material. A 70 μL aliquot the aqueous layer was dried down in a speedvac at room temperature for 4 h. Two derivatization steps (oximation and trimetylsilylation) were performed by a robotic Gerstel MPS2 multipurpose sampler (GERSTEL GmbH & Co. KG, Mülheim an der Ruhr, Germany) in which dried extracts were incubated at 37 °C for 90 min after adding 10 μL of anhydrous pyridine (Sigma-Aldrich, St. Louis, MO, USA) containing 20 mg/mL methoxyamine hydrochloride (Sigma-Aldrich, St. Louis, MO, USA). Subsequently, 15μL of *N*-methyl-*N*-(trimethylsilyl) trifluoroacetamide (MSTFA; Sigma-Aldrich, St. Louis, MO, USA) was added to the samples and incubated at 37 °C for 30 min. For Kovats non-isothermal RI matching to confirm identification of metabolites, n-alkanes (5 μL of 29 mg/L, C_12_-C_36_, Sigma-Aldrich, St. Louis, MO, USA) was also added to the samples and incubated for 1 min at 37 °C before injection into the GC-MS for analysis. 

A single quadrupole GC/MSD instrument (Agilent Technologies, Palo Alto, CA, USA) was used in this study. Samples of 1μL were injected in splitless mode into the GC (Agilent 7890A) that was coupled to a single quadrupole mass spectrometer (Agilent 5975C). The GC was equipped with a J&W VF-5 ms column (30 m × 0.25 mm × 0.25 μm), equipped with a 10 m EZ-Guard column (Agilent Technologies, Palo Alto, CA, USA). The injector temperature was 230 °C and helium was used as a carrier gas at a flowrate of 1 mL/min. Oven was held at an initial temperature of 70 °C for 1 min and increased to 325 °C at a ramp rate of 15 °C/min, then held at 325 °C for 3 min, making a total run time of 21 min. The electron impact (EI) ion source and quadrupole were kept at 250 °C and 150 °C, respectively. The filament current was set at 70 eV. The auxiliary transfer line was at 260 °C and the quadrupole mass analyser was operated in full MS scan acquisition mode from 40–600 *m/z* using a scan rate of 3.6 Hz. Solvent delay was 5.60 min. A QC sample was prepared by pooling aliquots from all sample extracts. The QC sample was injected several times at the start of the run and also after every 8 samples until the end of the batch sequence. Blank samples were run at the start and end of the batch and all samples were run in a randomized order.

### 4.6. Microarray Analysis

Microarray analysis was performed by the Ramaciotti Centre for Gene Function Analysis. Mouse intestinal RNA was isolated using a Nucleospin RNA plus kit (Macherey Nagel, Düren, Germany). RNA quality was measured with an Agilent Bioanalyser system and only RNA with an integrity >7.0 was used for RNA analysis on an Agilent 60K mouse GE array.

### 4.7. Data Analysis

Agilent MSD Chemstation software (version E.02) was used for data acquisition. The acquired MS data was converted into CDF format by Agilent MSD Chemstation software (Agilent Technologies, Palo Alto, CA, USA). XCMS Online was used for the pre-treatment of the data using GC/Single Quad “Matched Filter” settings which included automated peak picking, baseline filtering, peak grouping and retention time correction if needed [61,62]. No alterations were made to the default settings of the data processing. Mass spectral features along with retention time were retrieved in a “.csv” file along with their peak intensities. The internal standard (ribitol) was used to check the reproducibility of its peak area and retention time in each sample. Features associated with the internal standard and those present in the blank samples or coming from the column were removed from the data set. Then QC samples were also used to remove features that showed a relative standard deviation of more than 40 percent [34]. Features that are coming from the same metabolite were clustered together by CAMERA function of XCMS Online. Generally, the most intense ion of a feature that did not co-elute with an adjacent feature to give a pure annotated peak was selected as the quantifier ion and the remaining fragment ions of that metabolite were removed from the dataset. After data filtration, AMDIS (Automated Mass Spectral Deconvolution & Identification System) was used to annotate features by comparing their mass spectrum to those deposited in the GOLM database [63] and the National Institute of Standards and Technology (NIST) database. A feature was annotated as a metabolite by comparing the mass spectrum and RI of that peak with the reference mass spectrum and Kovats RI library in the GOLM database. The match was considered confident if the retention index difference was <3 RI and the mass spectrum similarity had a score >850. If a feature was not identified in the GOLM database, it was further searched in NIST and was annotated if it had a reverse spectral match of >700. Peak area was used as a measure of relative quantification of metabolites using the quantifier ion characteristic of that metabolite. Raw metabolomics dataset is uploaded on MetabolomeExpress [59] whereas metadata is attached as a supplementary file.

### 4.8. Statistical Analysis

After filtration, data was subjected to univariate and multivariate analysis to calculate fold-changes between ko and wt mice samples. All metabolites with fold-change >1.5 and *p* value less than 0.05 were selected. The most significant metabolites that differed in their abundance between samples were used to create a HCA and heat map using MetaboAnalyst 4.0 [64]. The data was log transformed to create a principal component analysis (PCA). PCA was used to visualize the clustering difference between metabolic profiles of all sample groups. The statistical significance was determined using Mann-Whitney and Dunn’s multiple comparison test using GraphPad Prism v5.01 (GraphPad software, La Jolla, CA, USA). Receiver operating characteristics were constructed to evaluate the potential of biomarkers. The ROC curves were validated by permutation testing. The top candidates for the ROC curve were selected using MetaboAnalyst. 

## Figures and Tables

**Table 1:** Body weight, urea levels in plasma and urine of SLC6A19ko and SLC6A19wt Mice.

	Genotype	Body Weight (g)	Urea (Plasma) mmol/L	Urea (Urine) mmol/L	Ammonia (Plasma) mg/mL	Ammonia (Urine) µg/mL	Ammonia (Faeces) mg/mL
Females	ko	26.1 ± 2.5	6.5 ± 1.5	568 ± 23.6	0.7 ± 0.2	7.5 ± 0.2	11.2 ± 2.8
	wt	29.4 ± 5.2	8.5 ± 2.1	423 ± 56.8	0.7 ± 0.1	7.4 ± 0.1	5.8 ± 1.5
Males	ko	22.9 ± 4.2	7.1 ± 2.6	618 ± 30.3	0.3 ± 0.4	4.9 ± 0.1	9.2 ± 1.8
	wt	27.9 ±4.6	8.2 ± 2.9	566 ± 19.9	0.3 ± 0.1	4.8 ± 0.2	3.3 ± 0.8

**Table 2:** Metabolites in the urine of SLC6A19ko and SLC6A19wt mice.

Metabolite	RI	Quant Ion (*m*/*z*)	Retention Time (min)	ko/wt		Females		Males		QC %RSD	ID Level
				FC	*p* Value	FC	*p* Value	FC	*p* Value		
Serine	1350	204.1	7.82	211.44	1.95 × 10^−7^	173.01	1.97 × 10^−4^	264.67	9.31 × 10^−4^	11.6	1
Isoleucine	1288	158.1	7.27	194.89	6.21 × 10^−8^	148.17	1.02 × 10^−4^	258.11	4.17 × 10^−4^	13.3	1
Threonine	1375	291.2	8.04	192.66	1.84 × 10^−9^	178.06	1.77 × 10^−5^	213.14	3.99 × 10^−4^	8.84	1
Leucine	1261	158.1	7.05	191.17	2.17 × 10^−8^	195.86	2.06 × 10^−5^	219.21	9.81 × 10^−5^	33.4	1
Methionine	1515	176.1	9.25	184.73	1.05 × 10^−8^	173.84	4.87 × 10^−5^	196.27	6.61 × 10^−4^	9.4	1
Glutamine	1723	156.0	10.81	146.47	3.63 × 10^−11^	228.76	1.19 × 10^−6^	121.57	2.09 × 10^−4^	12.1	1
Valine	1206	144.1	6.57	107.94	1.82 × 10^−7^	81.72	4.17 × 10^−5^	143.93	7.04 × 10^−4^	10.5	1
Glycine	1303	174.1	7.41	91.06	1.06 × 10^−8^	71.84	4.41 × 10^−6^	118.73	4.38 × 10^−5^	25.5	1
Phenylalanine	1630	192.1	10.13	84.99	4.24 × 10^−7^	57.00	5.02 × 10^−5^	123.75	6.26 × 10^−4^	9.0	1
Tyrosine	1931	218.1	12.34	70.60	2.04 × 10^−8^	61.11	3.35 × 10^−5^	83.72	6.62 × 10^−4^	9.9	1
Proline	1298	142.1	7.28	57.90	2.34 × 10^−6^	57.05	4.11 × 10^−4^	63.12	3.40 × 10^−3^	35.7	1
Aminobutyric acid	1161	130.1	6.17	56.53	3.81 × 10^−8^	31.51	1.03 × 10^−4^	120.84	1.11 × 10^−3^	9.5	1
Asparagine	1668	231.1	10.40	42.83	2.29 × 10^−9^	33.20	2.73 × 10^−4^	53.77	3.84 × 10^−4^	6.4	1
Cystine	2289	266.1	14.51	36.43	3.56 × 10^−4^	22.89	5.40 × 10^−3^	54.72	1.04 × 10^−2^	30.8	2
Nitrophenyl Glucuronide	2344	375.2	14.80	35.02	2.99 × 10^−6^	30.66	2.61 × 10^−4^	43.74	2.19 × 10^−3^	7.9	3
Pyroglutamate	1523	156.0	9.31	32.84	2.00 × 10^−15^	30.31	7.63 × 10^−9^	35.14	4.62 × 10^−6^	13.6	2
Tryptophan	2216	202.1	14.13	26.01	2.82 × 10^−7^	29.54	3.95 × 10^−4^	26.53	1.50 × 10^−3^	16.2	2
Aminomalonic acid	1461	320.1	8.79	17.40	1.19 × 10^−2^	10.32	1.90 × 10^−2^	27.11	5.15 × 10^−3^	27.6	2
Alanine	1360	262.1	7.83	17.39	8.68 × 10^−7^	10.94	6.45 × 10^−4^	27.21	9.58 × 10^−4^	14.2	2
Ornithine	1609	142.1	9.96	17.05	7.26 × 10^−7^	11.50	3.04 × 10^−4^	24.80	1.17 × 10^−3^	31.7	2
Phenaceturic acid	1896	250.1	12.10	14.05	1.48 × 10^−8^	10.33	4.10 × 10^−5^	18.88	4.06 × 10^−4^	10.0	3
Asparagine ester	1618	216.1	10.03	12.80	4.86 × 10^−4^	16.42	1.98 × 10^−2^	12.93	3.24 × 10^−2^	31.1	2
Cresol glucuronide	2405	375.2	15.21	8.80	9.91 × 10^−6^	5.51	8.41 × 10^−4^	13.93	1.75 × 10^−3^	5.9	3
Glutamic acid	1614	246.1	9.99	7.38	2.94 × 10^−7^	5.74	1.49 × 10^−5^	9.44	6.22 × 10^−3^	9.6	1
5 Hydroxy Indole	1719	277.1	10.79	5.89	1.02 × 10^−7^	5.70	2.88 × 10^−5^	5.79	6.50 × 10^−3^	26.1	2
Lysine	1908	230.1	12.19	5.18	3.24 × 10^−4^	3.50	2.19 × 10^−3^	7.49	1.74 × 10^−2^	6.3	1
Histidine	1915	154.1	12.23	4.05	6.41 × 10^−5^	5.71	1.40 × 10^−3^	3.18	2.66 × 10^−2^	31.9	1
Glutaric acid 3 hydroxy	1596	247.1	9.86	2.82	2.01 × 10^−3^	2.30	2.05 × 10^−2^	3.17	1.27 × 10^−1^	12.7	2
Serine N-acetyl	1499	261.1	9.13	2.22	2.30 × 10^−3^	1.82	1.74 × 10^−2^	2.86	1.10 × 10^−2^	11.1	2
Adipic acid	1498	186.0	9.13	2.19	2.79 × 10^−3^	1.94	1.33 × 10^−2^	2.70	1.46 × 10^−2^	8.7	2
Alanine beta	1423	174.1	8.46	1.65	4.35 × 10^−2^	1.10	8.15 × 10^−1^	2.48	3.17 × 10^−2^	34.3	1
3hydroxyphenyl acetic acid	1614	164.0	9.98	1.63	1.27 × 10^−2^	1.38	3.18 × 10^−1^	1.90	3.30 × 10^−2^	13.1	2
Putrescine	1739	174.1	10.93	0.98	9.64 × 10^−1^	2.36	7.55 × 10^−3^	1.12	8.10 × 10^−1^	12.5	1
Succinic acid	1311	247.1	7.47	0.73	2.00 × 10^−1^	0.80	5.86 × 10^−1^	0.54	1.80 × 10^−2^	20.8	1
Quinolone 2 carboxylic acid	2262	406.2	14.37	0.55	1.17 × 10^−2^	0.57	1.50 × 10^−1^	0.62	3.86 × 10^−2^	21.9	2
Butyrylglycine	1447	158.1	8.66	0.52	1.12 × 10^−2^	0.38	6.22 × 10^−3^	0.70	8.70 × 10^−2^	15.8	2
Thymine	1397	270.1	8.24	0.48	5.57 × 10^−3^	0.47	1.46 × 10^−1^	0.56	4.23 × 10^−2^	30.2	2
Hexanoylglycine	1644	158.1	10.23	0.39	2.96 × 10^−3^	0.40	1.26 × 10^−2^	0.45	1.72 × 10^−2^	22.9	2
Allantoin	1876	188.0	11.96	0.26	5.08 × 10^−3^	0.19	7.09 × 10^−2^	0.37	4.23 × 10^−2^	29.2	2
Taurine	1672	326.1	10.44	0.24	6.57 × 10^−3^	0.28	1.84 × 10^−1^	0.13	2.07 × 10^−3^	14.7	2

Fold changes (FC) are listed by genotype and gender. Metabolite ID levels indicate the confidence of identification, where 1: Confirmed identification with standard, 2: Putative identification with base peak (Quant) ion (*m*/*z*) and Kovats retention index (RI), 3: NIST reference spectra match score of greater than 700.

**Table 3:** Microarray analysis of other SLC transporters present in kidney.

Transporter	Acronym	SLC6A19ko	SLC6A19wt	Fold Change (ko/wt)	*p* Value
SLC7A9	b^0,+^AT	14.1	14	1.1	0.5
SLC7A7	y+LAT1/4F2hc	14.1	15	0.94	0.06
SLC1A1	EAAT3	12.9	13.5	0.96	0.05
SLC3A1	rBAT	14.3	14.5	0.99	0.37

**Table 4:** Metabolites changes in the faeces of SLC6A19ko and SLC6A19wt mice.

Metabolite ID	RI	Quant Ion (m/z)	Retention Tine (min)	ko/wt		Females		Males		QC %RSD	ID Level
				FC	*p* Value	FC	*p* Value	FC	*p* Value		
Acetylglutamine	1720	227.07	10.81	25.86	1.34 × 10^−2^	37.20	2.26 × 10^−2^	7.29	4.34 × 10^−2^	40.2	3
Aminobutanoic acid	1163	130.05	6.15	24.46	1.60 × 10^−5^	31.85	1.14 × 10^−3^	18.80	1.23 × 10^−2^	11.9	1
Leucine	1264	158.10	7.05	20.34	1.55 × 10^−5^	27.80	5.86 × 10^−4^	13.35	2.01 × 10^−2^	9.3	1
Norvaline	1230	144.05	6.76	16.67	1.85 × 10^−6^	17.77	1.97 × 10^−4^	14.70	6.58 × 10^−3^	8.4	3
Valine	1207	144.10	6.55	13.98	9.24 × 10^−5^	17.99	1.74 × 10^−3^	9.94	4.13 × 10^−2^	6.7	1
Putrescine	1740	174.04	10.93	11.90	6.43 × 10^−3^	16.48	2.64 × 10^−2^	6.58	1.18 × 10^−1^	15.7	1
Phenylalanine	1633	192.07	10.13	11.52	6.21 × 10^−5^	16.57	8.63 × 10^−4^	7.44	5.16 × 10^−2^	13.5	1
Dihydroxyphenyl-alanine	2086	368.05	13.32	11.37	9.60 × 10^−6^	9.41	1.38 × 10^−3^	17.68	2.63 × 10^−3^	39.8	3
Isoleucine	1287	158.10	7.25	10.07	2.43 × 10^−4^	12.81	3.10 × 10^−3^	7.37	5.94 × 10^−2^	10.8	1
Tyrosine	1931	218.08	12.34	9.63	3.06 × 10^−3^	10.78	6.90 × 10^−3^	8.56	1.51 × 10^−1^	19.1	1
Tryptophan	2216	202.06	14.13	7.99	2.65 × 10^−3^	10.25	2.71 × 10^−2^	5.72	4.38 × 10^−2^	34.8	1
Glycine	1303	174.06	7.39	6.89	3.49 × 10^−3^	8.42	2.76 × 10^−2^	5.67	8.87 × 10^−2^	13.8	1
Tyramine	1912	338.03	12.21	6.20	1.42 × 10^−3^	10.40	3.05 × 10^−2^	4.40	1.35 × 10^−2^	15.2	2
Methionine sulfoxide	1782	128.00	11.25	5.98	1.25 × 10^−3^	7.56	2.49 × 10^−2^	4.52	8.16 × 10^−3^	9.9	2
Homoserine	1441	218.10	8.60	5.79	4.49 × 10^−4^	7.56	4.30 × 10^−3^	3.56	2.66 × 10^−2^	39.3	2
Homocystine ester	1857	128.00	11.80	5.62	3.14 × 10^−3^	7.26	3.42 × 10^−2^	3.99	1.11 × 10^−2^	11.3	2
5 amino valeric acid	1633	174.07	10.11	4.44	4.11 × 10^−6^	5.70	7.35 × 10^−5^	3.14	3.38 × 10^−2^	9.1	2
3 hydroxyphenyl-acetic acid	1613	163.97	9.98	4.05	2.02 × 10^−4^	4.26	9.13 × 10^−3^	3.72	2.59 × 10^−3^	12.5	2
Threonine	1377	291.09	8.03	3.68	2.69 × 10^−2^	4.45	1.02 × 10^−1^	2.81	1.18 × 10^−1^	18.7	1
Ornithine	1810	142.02	11.46	3.59	2.41 × 10^−3^	4.79	5.85 × 10^−3^	1.97	2.08 × 10^−1^	4.9	1
Phenylacetic acid	1302	163.99	7.41	3.12	5.82 × 10^−4^	4.26	2.26 × 10^−3^	2.13	1.45 × 10^−1^	20.2	3
Lysine	1908	174.05	12.19	2.96	1.03 × 10^−2^	4.20	3.53 × 10^−2^	1.75	8.27 × 10^−2^	13.1	1
Serine	1352	204.08	7.81	2.94	3.34 × 10^−2^	3.84	9.41 × 10^−2^	2.16	2.35 × 10^−1^	15.8	1
Succinic acid	1309	247.02	7.45	2.66	6.86 × 10^−6^	3.39	2.22 × 10^−4^	1.96	2.40 × 10^−2^	20.7	1
Indole 5 hydroxy	1791	277.05	11.32	2.50	6.51 × 10^−4^	2.95	2.48 × 10^−3^	1.96	1.39 × 10^−1^	14.8	2
Proline	1297	142.06	7.34	2.09	7.96 × 10^−3^	2.49	1.04 × 10^−2^	1.79	2.15 × 10^−1^	14.8	1
4 hydroxyphenyl-acetic acid	1643	163.98	10.23	2.08	6.74 × 10^−4^	1.66	4.33 × 10^−2^	2.89	1.36 × 10^−2^	16.6	2
Alanine	1137	116.03	5.86	1.88	2.92 × 10^−3^	1.82	2.63 × 10^−2^	1.96	8.15 × 10^−2^	31.1	1
Myoinositol	2077	318.09	13.26	1.88	2.49 × 10^−2^	1.86	1.73 × 10^−1^	1.93	2.24 × 10^−2^	17.3	1
Threitol	1488	128.01	9.03	1.60	2.45 × 10^−2^	1.85	2.84 × 10^−2^	1.40	2.84 × 10^−1^	14.7	2
Valero 1,5-lactam	1143	156.00	6.01	1.43	1.21 × 10^−2^	1.76	1.18 × 10^−3^	1.10	7.14 × 10^−1^	32.5	2
Glyceric acid	1318	292.06	7.54	1.35	2.27 × 10^−1^	1.77	2.07 × 10^−2^	1.07	8.71 × 10^−1^	40.2	2
Benzoic acid	1254	178.98	6.97	1.11	3.98 × 10^−2^	1.03	5.37 × 10^−1^	1.24	4.56 × 10^−3^	18.1	2
alpha tocopherol	3155	502.41	18.76	0.85	2.70 × 10^−1^	0.59	1.34 × 10^−2^	1.51	2.15 × 10^−2^	13.6	2
1-monoleoylglycerol	2748	397.33	16.39	0.79	3.35 × 10^−2^	0.66	1.44 × 10^−2^	0.99	9.32 × 10^−1^	10.8	2
Sitosterol	3371	357.32	19.71	0.78	2.80 × 10^−2^	0.66	1.08 × 10^−2^	1.01	9.41 × 10^−1^	14.2	3
Stigmastan-3-ol	3386	215.11	19.77	0.77	2.59 × 10^−2^	0.63	3.49 × 10^−3^	1.08	5.77 × 10^−1^	14.3	3
Heptdecanoic acid	2141	327.24	13.68	0.76	1.67 × 10^−2^	0.64	1.45 × 10^−3^	0.94	6.35 × 10^−1^	23.8	3
Tetradecanoic acid	1845	285.20	11.75	0.74	1.30 × 10^−2^	0.64	1.05 × 10^−2^	0.90	3.97 × 10^−1^	35.9	2
Allose	1914	299.20	12.23	0.69	1.12 × 10^−2^	0.54	5.79 × 10^−3^	0.90	5.42 × 10^−1^	37.4	2
Campesterol	3291	343.28	19.35	0.46	3.96 × 10^−4^	0.43	8.48 × 10^−3^	0.50	3.42 × 10^−2^	15.1	3
Deoxycholic acid	3296	266.10	19.39	0.41	1.63 × 10^−2^	0.24	1.06 × 10^−2^	1.08	7.90 × 10^−1^	20.4	3
Sebacic acid	2424	215.12	15.22	0.33	6.33 × 10^−4^	0.19	1.37 × 10^−3^	0.60	2.53 × 10^−1^	8.6	3

Fold changes are listed by genotype and gender. Metabolite ID levels indicate the confidence of identification, where 1: Confirmed identification with standard, 2: Putative identification with base peak (Quant) ion (*m*/*z*) and Kovats retention index (RI), 3: NIST reference spectra match score of greater than 700.

**Table 5:** Metabolites changes in the plasma of SLC6A19ko and SLC6A19wt mice.

Metabolite ID	RI	Quant Ion (*m*/*z*)	Retention Time (min)	ko/wt		Females		Males		QC %RSD	ID Level
				FC	*p* Value	FC	*p* Value	FC	*p* Value		
Unknown	2606	254.1	16.07	23.59	3.65 × 10^−2^	10.39	1.06 × 10^−2^	29.42	6.05 × 10^−2^	13.7	4
p-cresol glucuronide	2343	375.1	14.70	17.34	9.88 × 10^−3^	14.86	2.63 × 10^−2^	19.37	1.03 × 10^−1^	33.7	3
Indole 3 propionic acid	2095	202.1	13.28	4.41	2.19 × 10^−2^	4.46	1.74 × 10^−2^	4.39	1.01 × 10^−1^	12.2	2
Phenaceturic acid	1895	250.1	12.02	2.92	4.68 × 10^−4^	2.41	2.37 × 10^−3^	3.77	4.51 × 10^−2^	19.8	3
Proline	1298	142.1	7.28	2.50	9.79 × 10^−3^	2.14	4.71 × 10^−2^	2.68	8.88 × 10^−3^	12.5	1
Xanthine	2017	353.1	12.79	1.65	9.01 × 10^−2^	0.80	5.87 × 10^−1^	3.99	2.38 × 10^−3^	10.7	2
Histidine	1914	254.1	12.14	1.47	3.17 × 10^−2^	1.21	2.19 × 10^−1^	1.67	1.18 × 10^−2^	14.4	1
Malate	1474	233.1	8.83	1.47	4.16 × 10^−2^	1.33	3.54 × 10^−1^	1.57	2.51 × 10^−2^	11.6	1
Glutamic acid	1613	246.1	9.90	1.43	9.44 × 10^−2^	0.89	7.04 × 10^−1^	2.12	6.80 × 10^−3^	7.2	1
Lysine	1907	174.1	12.11	1.42	1.07 × 10^−3^	1.29	1.77 × 10^−3^	1.55	3.12 × 10^−2^	16.7	1
Ornithine	1809	142.1	11.37	1.38	6.81 × 10^−2^	1.34	7.76 × 10^−3^	1.41	1.54 × 10^−1^	14.8	1
Glutamine	1771	156.1	11.09	1.36	2.65 × 10^−2^	1.08	6.17 × 10^−1^	1.65	1.75 × 10^−2^	7.3	1
Myoinositol	2009	305.1	12.75	1.28	3.29 × 10^−2^	1.05	6.16 × 10^−1^	1.52	1.88 × 10^−2^	14.1	1
Serine	1350	218.1	7.74	1.25	6.11 × 10^−2^	1.11	4.09 × 10^−1^	1.37	4.94 × 10^−2^	13.5	1
Fumaric acid	1347	245.1	7.70	1.16	4.80 × 10^−1^	0.60	6.17 × 10^−2^	1.73	4.80 × 10^−3^	13.9	1
Tyrosine	1931	218.1	12.25	1.15	2.69 × 10^−1^	1.05	5.44 × 10^−1^	1.24	2.49 × 10^−1^	8.1	1
Phenylalanine	1632	192.1	10.04	1.14	4.24 × 10^−1^	1.18	1.68 × 10^−1^	1.11	5.73 × 10^−1^	10.9	1
Stearic acid	2242	341.3	14.16	0.95	4.59 × 10^−1^	0.81	2.63 × 10^−2^	1.08	3.09 × 10^−1^	7.2	1
Octadecanol	2143	327.2	13.58	0.92	3.47 × 10^−1^	0.77	1.20 × 10^−2^	1.04	6.70 × 10^−1^	18.1	2
Leucine	1265	158.2	6.99	0.90	4.80 × 10^−1^	1.09	6.24 × 10^−1^	0.79	9.38 × 10^−2^	14.1	1
Valine	1208	144.1	6.49	0.86	3.00 × 10^−1^	0.96	7.71 × 10^−1^	0.80	1.05 × 10^−1^	13.2	1
Isoleucine	1288	158.2	7.19	0.81	1.42 × 10^−1^	0.94	6.46 × 10^−1^	0.73	5.65 × 10^−2^	13.4	1
Glucose	1876	217.1	11.88	0.78	4.77 × 10^−4^	0.72	1.59 × 10^−2^	0.85	3.54 × 10^−3^	8.7	1
Uric acid	2088	441.2	13.24	0.77	8.27 × 10^−2^	0.94	7.73 × 10^−1^	0.67	1.02 × 10^−2^	8.7	1
Threonine	1376	291.1	7.96	0.73	1.65 × 10^−2^	0.69	5.60 × 10^−2^	0.76	8.27 × 10^−2^	16.5	1
Glycerol	1262	205.1	6.96	0.70	4.83 × 10^−3^	0.60	3.69 × 10^−3^	0.81	2.82 × 10^−1^	9.2	1
Palmitic acid	2046	313.2	12.97	0.69	7.69 × 10^−3^	0.55	2.10 × 10^−2^	0.84	3.14 × 10^−1^	11.1	1
Linoleic acid	2210	337.2	14.00	0.67	2.32 × 10^−2^	0.52	1.85 × 10^−2^	0.81	3.61 × 10^−1^	10.1	1
Tryptophan	2215	202.1	14.03	0.64	4.44 × 10^−4^	0.47	1.10 × 10^−3^	0.84	1.32 × 10^−1^	12.1	1
Cysteine	1550	220.1	9.42	0.63	1.8 × 10^−2^	0.71	1.10 × 10^−1^	0.58	4.76 × 10^−2^	24.1	1
11-cis-Octadecenoic acid	2215	339.2	14.03	0.43	3.24 × 10^−3^	0.35	2.43 × 10^−2^	0.54	1.19 × 10^−1^	9.9	2
Oleic acid	2748	397.3	16.82	0.36	2.88 × 10^−3^	0.44	9.66 × 10^−2^	0.29	2.81 × 10^−2^	18.9	2
cis-9-Hexadecenoic acid	2026	311.2	12.85	0.19	1.10 × 10^−2^	0.16	2.56 × 10^−^2	0.22	2.05 × 10^−1^	10.2	2

Fold changes are listed by genotype and gender. Metabolite ID levels indicate the confidence of identification, where 1: Confirmed identification with standard, 2: Putative identification with base peak (Quant) ion (m/z) and Kovats retention index (RI), 3: NIST reference spectra match score of greater than 700, and 4: unknown.

**Table 6:** AUROC analysis of metabolites in urine, faeces and plasma to distinguish between SLC6A19ko and SLC6A19wt mice.

Urine			Faeces			Plasma		
ID	AUC	*p* Value	ID	AUC	*p* Value	ID	AUC	*p* Value
**Aminobutyric acid**	1	8.80 × 10^−10^	Aminobutyric acid	1	1.2 × 10^−6^	Indole 3 Propionic acid	0.99	1.32 × 10^−2^
**Valine**	1	5.50 × 10^−9^	Valine	1	1.50 × 10^−5^	p−cresol glucuronide	0.99	4.38 × 10^−3^
**Isoleucine**	1	3.00 × 10^−11^	Leucine	1	9.80 × 10^−7^	Ornithine	0.95	2.15 × 10^−3^
**Leucine**	1	5.60 × 10^−10^	5−aminovalerate	1	2.50 × 10^−7^	Tryptophan	0.94	4.29 × 10^−4^
**Glycine**	1	1.40 × 10^−9^	Phenylalanine	0.992	2.40 × 10^−6^	11Octadecenoic acid	0.93	1.22 × 10^−3^
**Serine**	1	4.40 × 10^−9^	Tryptophan	0.975	1.90 × 10^−3^	Glucose	0.92	4.28 × 10^−4^
**Threonine**	1	5.30 × 10^−12^	Isoleucine	0.975	6.40 × 10^−5^	Phenaceturic acid	0.91	1.67 × 10^−4^
**Methionine**	1	1.80 × 10^−10^	Tyrosine	0.975	0.001948	Tetradecanoic acid	0.91	5.41 × 10^−3^
**Phenylalanine**	1	1.40 × 10^−8^	Dihydro × yphenylalanine	0.975	1.37 × 10^−6^	Lysine	0.9	9.17 × 10^−4^

## Supplementary Materials

Supplementary materials and paper metadata can be found at http://www.mdpi.com/1422-0067/19/11/3597/s1.

## Abbreviations

ACE2	Angiotension converting enzyme 2
AUC	Area under curve
B^0^AT1	Broad neutral amino acid transporter
BCAA	Branched chain amino acids
FGF21	Fibroblast growth factor 1
GC-MS	Gas chromatography mass spectrometry
GCN2	General control non derepressible 2
GLP-1	Glucagon like peptide 1
GMD	Golm Metabolome Database
HCA	Heirarchial clustering analysis
mTORC1	Mammalian target of rapamycin 1
NIST	National Institute of Standards and Technology
NZO	Newzealand obese
PCA	Principal component analysis
QC	Quality control
RI	Retention index
ROC	Receiver operating characteristic
