# Supplementary material for: Development of Biomarkers for Inhibition of SLC6A19 (B0AT1)—A Potential Target to Treat Metabolic Disorders
Source: Int J Mol Sci. 2018 Nov 14;19(11):3597. doi: 10.3390/ijms19113597 (PMC6274964; doi:10.3390/ijms19113597)
Supplement: Supplementary file 1 [file ijms-19-03597-s001.zip › Supplementary files/Supplementry table.docx]

**Suppl.Table 1.** Composition of the standard chow diet used in the study

| **RAT AND MOUSE PREMIUM BREEDER DIET 23% PROTEIN** | | | |
| --- | --- | --- | --- |
| **Ingredients** | | | |
| Wheat | | | |
| Sorghum | | | |
| Soybean meal | | | |
| Pollard | | | |
| Bran | | | |
| Meat and bone meal | | | |
| Bloodmeal | | | |
| Fish meal | | | |
| Lucerne meal | | | |
| Vegetable oil | | | |
| Sunflower meal | | | |
| Salt | | | |
| Vitamin and mineral premix | | | |
| Lysine | | | |
| Choline chloride | | | |
| **Proximate Analysis of the Standard chow diet** | | | |
| Crude Protein | | 23% | |
| Crude Fat | | 4% | |
| Crude Fibre | | 5% | |
| M.E.(Min) | | 13mJ/kg | |
| **Amino Acids** |  | **Fats** |  |
| Lysine | 9.8g/kg | Saturated Fat | 21.30% |
| Methionine & Cystine | 5.9g/kg | Mono-unsaturated | 42.90% |
| Threonine | 8.4g/kg | Poly-unsaturated | 30.70% |
| Histidine | 5.0g/kg | **Vitamins** |  |
| Leucine | 15.2g/kg | Vitamin A | 170μg/100g |
| Arginine | 12.1g/kg | Vitamin B1 (Thiamine) | 4mg/kg |
| Valine | 10.2g/kg | Vitamin B2 (Riboflavin) | 5mg/kg |
| Isoleucine | 8.0g/kg | Vitamin B6 (Pyridoxine) | 6mg/kg |
| Phenylaline & Tyrosine | 16.4g/kg | Vitamin B12 (Cyanocobalamin) | 0.005mg/kg |
| Tryptophan | 3.7g/kg | Vitamin C (Ascorbic Acid) | 150mg/kg |
| Calcium | 10.1g/kg | Vitamin D | 200i.u/kg |
| Phosphorus | 7.7g/kg | Vitamin E (Tocopherol Acetate) | 50mg/kg |
| Potassium | 5.4g/kg | Vitamin K | 5mg/kg |
| Magnesium | 1.8g/kg | Niacin | 10mg/kg |
| Iron | 97.0mg/kg | Pantothenate | 12mg/kg |
| Copper | 10.6mg/kg | Folic Acid | 10mg/kg |
| Manganese | 87.4mg/kg | Biotin | 0.06mg/kg |
| Zinc | 48.1mg/kg |  |  |
| Iodine | 1.15mg/kg |  |  |
| Selenium | 0.1mg/kg |  |  |
| Sodium | 0.30% |  |  |

**Suppl. Table 2.** Concentration of amino acids used to prepare the unlabeled amino acid mix.

| **Amino acid** | **Weight (g)** |
| --- | --- |
| Arginine | 0.628 |
| Cysteine | 0.156 |
| Glycine | 0.512 |
| Histidine | 0.272 |
| Isoleucine | 0.424 |
| Leucine | 0.756 |
| Lysine | 0.592 |
| Methionine | 0.236 |
| Phenylalanine | 0.444 |
| Tyrosine | 0.3388 |
| Threonine | 0.388 |
| Tryptophan | 0.112 |
| Valine | 0.464 |
| Serine | 0.472 |
| Aspartic acid | 1.124 |
| Glutamic acid | 1.896 |
| Alanine | 0.576 |
| Proline | 0.588 |
| Taurine | 0.012 |
| Dissolved in 100ml of water | |

The unlabeled amino acid mixture was solidified using gelatin (14%wt/vol) kindly provided free of cost by Gelita Australia (QLD, Australia). 10ml of strawberry essence (Queen, Australia) was also added to the mixture to make it palatable for the mice. The amount of unlabeled amino acid mixture given to the mice was adjusted to 1g/kg of body weight of individual mice whereas labelled amino acid was kept constant for all mice.

**Suppl. Fig. 1.**

Mass spectral matches to p-cresol glucuronide (A) and indole-3-propionic acid (B)

**A)**

**
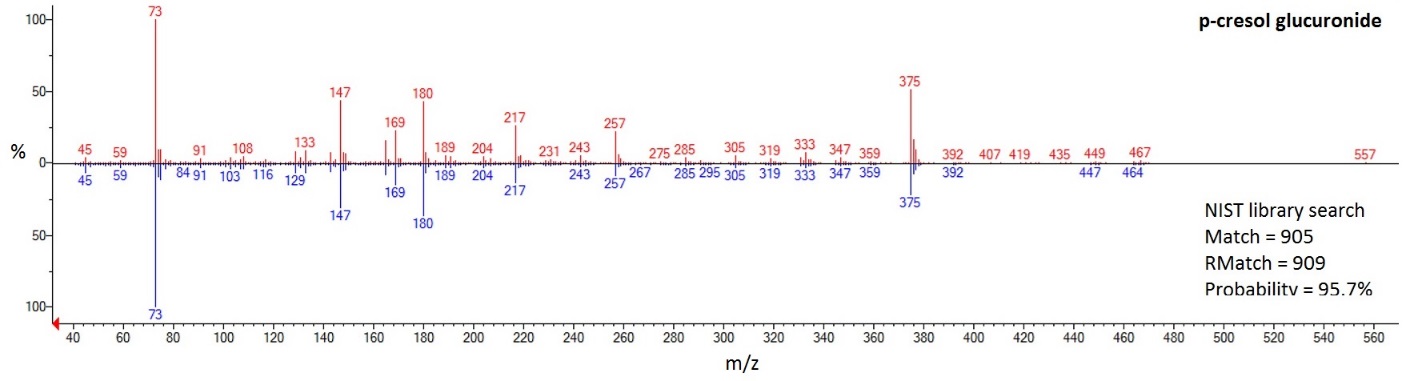
**

**B)**

**
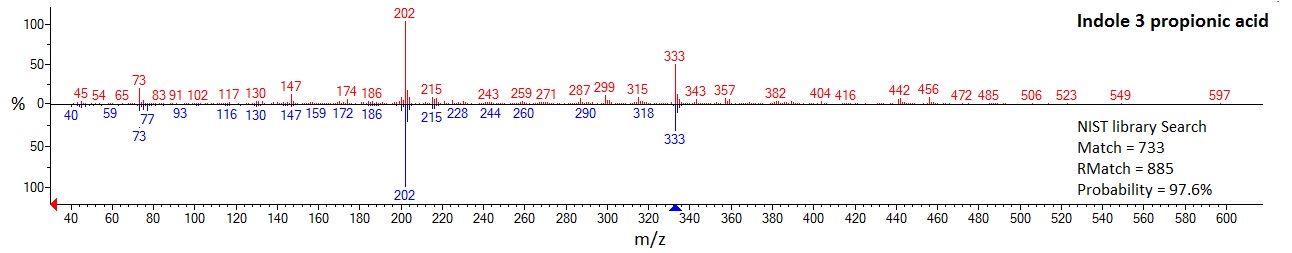
**
